# Real-world outcomes with ranibizumab in branch retinal vein occlusion: The prospective, global, LUMINOUS study

**DOI:** 10.1371/journal.pone.0234739

**Published:** 2020-06-18

**Authors:** Ian Pearce, Andreas Clemens, Michael H. Brent, Lin Lu, Roberto Gallego-Pinazo, Angelo Maria Minnella, Catherine Creuzot-Garcher, Georg Spital, Taiji Sakamoto, Cornelia Dunger-Baldauf, Ian L. McAllister, on behalf of all the LUMINOUS study investigators

**Affiliations:** 1 St Paul’s Eye Unit, Royal Liverpool University Hospital, Liverpool, England, United Kingdom; 2 Department of Eye and Vision Science, University of Liverpool, Liverpool, England, United Kingdom; 3 Novartis Pharma AG, Basel, Switzerland; 4 Department of Cardiology and Angiology I, Heart Center Freiburg University, Faculty of Medicine, University of Freiburg, Freiburg im Breisgau, Germany; 5 Department of Ophthalmology and Vision Sciences, Faculty of Medicine, University of Toronto, Ontario, Canada; 6 State Key Laboratory of Ophthalmology, Zhongshan Ophthalmic Center, Sun Yat-sen University, Guangzhou, China; 7 Unit of Macula, Oftalvist Clinic, Valencia, Spain; 8 Department of Ophthalmology, Catholic University of Sacred Heart - Foundation "Policlinico Universitario A. Gemelli"- IRCCS, Rome, Italy; 9 Department of Ophthalmology, Centre Hospitalier Universitaire, Dijon, France; 10 Department of Ophthalmology at St. Franziskus Hospital, Münster, Germany; 11 Department of Ophthalmology, Kagoshima University, Kagoshima, Japan; 12 Centre for Ophthalmology and Visual Science, The University of Western Australia, Perth, Australia; 13 Lions Eye Institute, The University of Western Australia, Perth, Australia; 14 Department of Ophthalmology, Royal Perth Hospital, Perth, Australia; University of Florida, UNITED STATES

## Abstract

**Objective:**

To evaluate the effectiveness, safety, and treatment patterns of ranibizumab 0.5 mg in treatment-naïve patients with branch retinal vein occlusion (BRVO) enrolled in the LUMINOUS^™^ study.

**Study design:**

A 5-year, global, prospective, multicenter, observational, open-label study conducted in a clinical practice (real-world) setting at outpatient ophthalmology clinics that recruited 30,138 consenting adult patients from all approved indications for ranibizumab across 42 countries. Patients with BRVO were treated according to the local ranibizumab label of the participating countries. Mean change in visual acuity (VA) in Early Treatment Diabetic Retinopathy Study letters from baseline to Year 1, treatment exposure during Year 1, and adverse events (AEs) over 5 years were assessed.

**Results:**

Of the 1366 recruited BRVO patients, 405 were treatment-naïve at baseline with a mean (standard deviation [SD]) age of 67.9 (12.5) years, 57.5% were female, and 71.8% were White. At Year 1 (n = 189), the mean (SD) VA gain was 11.9 (17.66) letters from a baseline of 49.2 (±20.32) letters with a mean (SD) of 5.0 (2.34) injections. VA gains were higher in patients (n = 83) who received 6–9 injections (13.6 [20.16] letters) than in those who received 2–5 injections (n = 92, 11.7 [15.43] letters), or 1 injection (n = 14, 3.6 [13.72] letters). Patients with baseline VA <23 letters had numerically highest VA gains (n = 20, 31.1 [24.48] letters). Over 5 years, the rate of ocular/non-ocular AEs was 7.4%/9.1% and serious AEs was 0.3%/4.4% in treatment-naïve BRVO patients (n = 405).

**Conclusions:**

One year results from the LUMINOUS real-world study showed a clinically meaningful VA improvement with ranibizumab in treatment-naïve patients with BRVO; numerically higher VA gains were achieved in patients who received more injections and those with poor baseline VA. No new safety signals were observed.

## Introduction

Branch retinal vein occlusion (BRVO) is one of the most common retinal vascular abnormality in adults and a frequent cause of vision loss [1]. The global prevalence of BRVO is ~0.64% [2]. Management with anti-vascular endothelial growth factor (anti-VEGF) agent therapy has become the current standard of care for most patients with BRVO [3, 4].

Ranibizumab (Lucentis^®^; Novartis Pharma AG, Basel, Switzerland, and Genentech Inc., South San Francisco, CA, USA), an anti-VEGF agent, is approved for the treatment of patients with macular edema secondary to retinal vein occlusion (RVO; BRVO and central retinal vein occlusion [CRVO]) in the European Union (EU), the United States (US), and many other countries worldwide [5, 6]. A wealth of scientific evidence from randomized clinical trials (RCTs) [4, 7–12] have proven the efficacy and safety of ranibizumab in the treatment of BRVO. These studies also indicated that early, intensive, and individualized treatment with ranibizumab provides visual acuity (VA) gains that are sustained over time with reduced treatment burden. Further, with over 5.5 million patient-treatment years of exposure [13] the efficacy and safety of ranibziumab is well-established across all approved indications [14–17]. However, real-world studies with ranibizumab in BRVO patients are mostly limited to either specific regions, countries or small patient populations [18–22]. Further, real-world evidence data coming from a more heterogenous patient population will help gain better insights on treatment patterns, outcomes, access to treatment, and management of BRVO patients with ranibizumab.

LUMINOUS (NCT01318941), the largest prospective observational trial in the field of medical retina, was conducted to evaluate the long-term effectiveness, safety, and treatment patterns with ranibizumab in routine clinical practice across five approved indications (neovascular age-related macular degeneration [nAMD], diabetic macular edema [DME], BRVO, CRVO, and myopic choroidal neovascularization [mCNV]) [23]. Here we report the 1-year VA outcomes, treatment patterns over one year, and overall safety over 5 years with ranibizumab in treatment-naïve patients with BRVO.

## Methods

### Study design

LUMINOUS was a 5-year, prospective, observational, global study of ranibizumab in patients with nAMD, DME, BRVO, CRVO, or mCNV. The study was conducted from March 2011 to April 2016 at 488 clinical sites across 42 countries [23]. Patients with BRVO were enrolled from 26 countries (S2 Table).

BRVO patients were treated with intravitreal ranibizumab 0.5 mg according to the local product label at outpatient ophthalmology clinics. As patients were recruited over time and the calendar time point of study completion was pre-set, follow-up time varied according to entry date. The minimum potential follow-up for each patient was defined as 1 year in the protocol. Visits took place at a frequency determined by the investigator; it was recommended to capture data at every visit or at a minimum of every 3 months. Physicians were encouraged to follow-up with patients who had not been seen in the clinic for at least 6 months. Patients not seen at least once per year or who were switched to another anti-VEGF therapy were discontinued from the study.

The first eye treated during the study was considered as the primary treated eye (study eye). If both eyes were first treated on the same date, or if both eyes were pre-treated, the eye with the earliest diagnosis date was considered the primary treated eye. If both eyes had the same diagnosis date, one eye was chosen randomly as the primary treated eye.

The study protocol was reviewed and approved by an Independent Ethics Committee (IEC) or Institutional Review Board (IRB) for each center. The complete list IEC or IRB by study center are available in S1 Table. The study was conducted in accordance with the Guidelines for Good Pharmacoepidemiology Practices issued by the International Society for Pharmacoepidemiology, with any applicable national guidelines, and ethical principles laid down in the Declaration of Helsinki. Patients provided written informed consent. The study is registered with clinicaltrials.gov as NCT01318941 [23].

### Key eligibility criteria

Consenting adult patients (≥18 years of age) enrolled were either treatment-naïve or previously treated with ranibizumab or another ocular therapy for any of the approved indications included in the local product label. Patients were excluded if they were participating in other investigational studies or if they received systemic or ocular anti-VEGF therapy other than ranibizumab 90 days or 30 days prior to enrollment, respectively.

### Assessments

Effectiveness assessments included VA (preferably best-corrected VA) evaluation by each participating physician as part of a routine care practice using Early Treatment Diabetic Retinopathy Study (ETDRS) letters, Snellen charts, or equivalent. To facilitate data analysis, Snellen fractions and decimals were converted to the ETDRS equivalent letter scores. It was recommended that the same method of corrected VA assessment be used throughout the study wherever possible. Other assessments, such as optical coherence tomography (i.e. central retinal thickness) and ocular examination (pre-injection intraocular pressure), were optional but included if the data were available. The number of ranibizumab injections administered overall, over time, and the average time interval (in weeks) between consecutive injections, visit frequency, treatment patterns, and proportion of patients receiving ocular and non-ocular concomitant medications were recorded. All adverse events (AEs), including serious AEs (SAEs), irrespective of suspected causal association that occurred during the study were collected.

### Statistical analysis

All effectiveness and safety data were summarized descriptively. The enrolled set included all patients who signed the informed consent, and had at least a baseline assessment. The safety set comprised patients who were treated with at least 1 dose of ranibizumab during the study or prior to the start of the study and had at least 1 safety assessment after the first treatment. The primary treated eye set included all primary treated eyes in patients from the safety set and was the primary analysis set for effectiveness. For Year 1 analysis, patients from the primary treated eye set who had both baseline and Year 1 data and had remained in the study for at least 365 days were considered. For treatment-naïve eyes, the date of first on-study injection with ranibizumab was considered the baseline date (study Day 1).

The primary effectiveness variable was the mean change in VA ETDRS letter score from baseline to Year 1. Additional effectiveness variables not prespecified in the protocol but included in the statistical analysis plan are: (1) The mean change in VA from baseline at Year 1 by (a) injection frequency during Year 1 (1, 2–5, 6–9 injections); (b) patients who received a loading dose (at least 3 ranibizumab injections up to Day 100) versus those who did not; (c) baseline VA category; (d) baseline VA of </≥73 letters (good starting vision, or Snellen equivalent 20/40) at Year 1; (2) the proportion of patients with VA loss (defined as ≤0 letter change from baseline) or gain (defined as >0 letter change from baseline) of >0 to<5 letters, 5 to <10 letters, 10 to <15 letters, and ≥15 letters at Year 1. The number of injections and visits up to 1 year were summarized for patients with at least 365 days participation in the study. Safety over the 5-year period was assessed based on the incidence, proportion, relationship, and severity of treatment-emergent ocular and non-ocular AEs. Ocular AEs were assessed for the primary treated eye set and non-ocular AEs were assessed for the safety set.

## Results

### Patient disposition and baseline characteristics

Overall 30,138 patients across all 5 indications were enrolled worldwide. Of these, 1366 (4.5%) were patients with BRVO, of whom 405 were treatment-naïve. Overall, 247 (61%) patients completed the 5-year study period, with the most frequent reason for study discontinuation being loss to follow-up (S3 Table). Of the 158 (39.01%) patients who discontinued the study at Year 5, only 19 (4.691%) patients switched to other anti-VEGF (S3 Table). Patients were recruited in 26 countries and most of the treatment-naïve patients with BRVO were recruited from the following 6 countries: the UK (n = 104), Canada (n = 68), Russia (n = 44), Germany (n = 38), India (28), and Spain (n = 21).

At 1 year, 326 (80.7%) treatment-naïve patients with BRVO remained in the LUMINOUS study. The most frequent reasons for study discontinuation in 78 (19.3%) patients were loss to follow-up (37 [9.16%]), followed by switching to an anti-VEGF other than ranibizumab (11 [2.72%)](S3 Table). As visits were scheduled at the discretion of the investigator, 1 Year data were available only for a proportion of the 326 patients. The primary treated eye set at Year 1 included 189 (46.7%) patients (qualified having both baseline and Year 1 data and remained in the study for at least 365 days) and the 5 year safety set included 405 patients.

Overall, the mean (standard deviation [SD]) age of patients was 67.9 (12.5) years at baseline; most were White (71.8%) and female (57.5%, Table 1). The proportion of patients with pre- and post-baseline laser therapy was 3.2% and 3.7%, respectively. The baseline patient and ocular characteristics of patients from each of these countries are described in S1 Fig. Country-level mean ages ranged from 58.1 (India) to 74.4 years (Canada). The country-level mean baseline VA ranges differed between countries and there was a large variation in median time from diagnosis to first treatment among countries, ranging from 1 (Canada) to 41 days (Russia).

**Table 1:** Baseline demographic and ocular characteristics (safety set).

Characteristics	Treatment-naïve patients with BRVO N = 405
Mean (SD) age, years	67.9 (12.5)
Gender, (%)	
Male	42.5
Female	57.5
Race, (%)	
White	71.8
Asian	12.6
Native American	1.5
Black	1.0
Other	5.4
VA	
n*	362
Mean (SD) VA, ETDRS letters	52.0 (19.5)
CRT	
n*	278
Mean (SD) CRT, μm	457.1 (159.3)
Median time from diagnosis to first treatment, days	18.0

*patients with evaluable data at baseline is shown

BRVO, branch retinal vein occlusion; CRT, central retinal thickness; EDTRS, Early Treatment Diabetic Retinopathy Study; N, total number of patients; n, number of patients; SD, standard deviation; VA, visual acuity

### Efficacy outcomes

**Fig 1:**
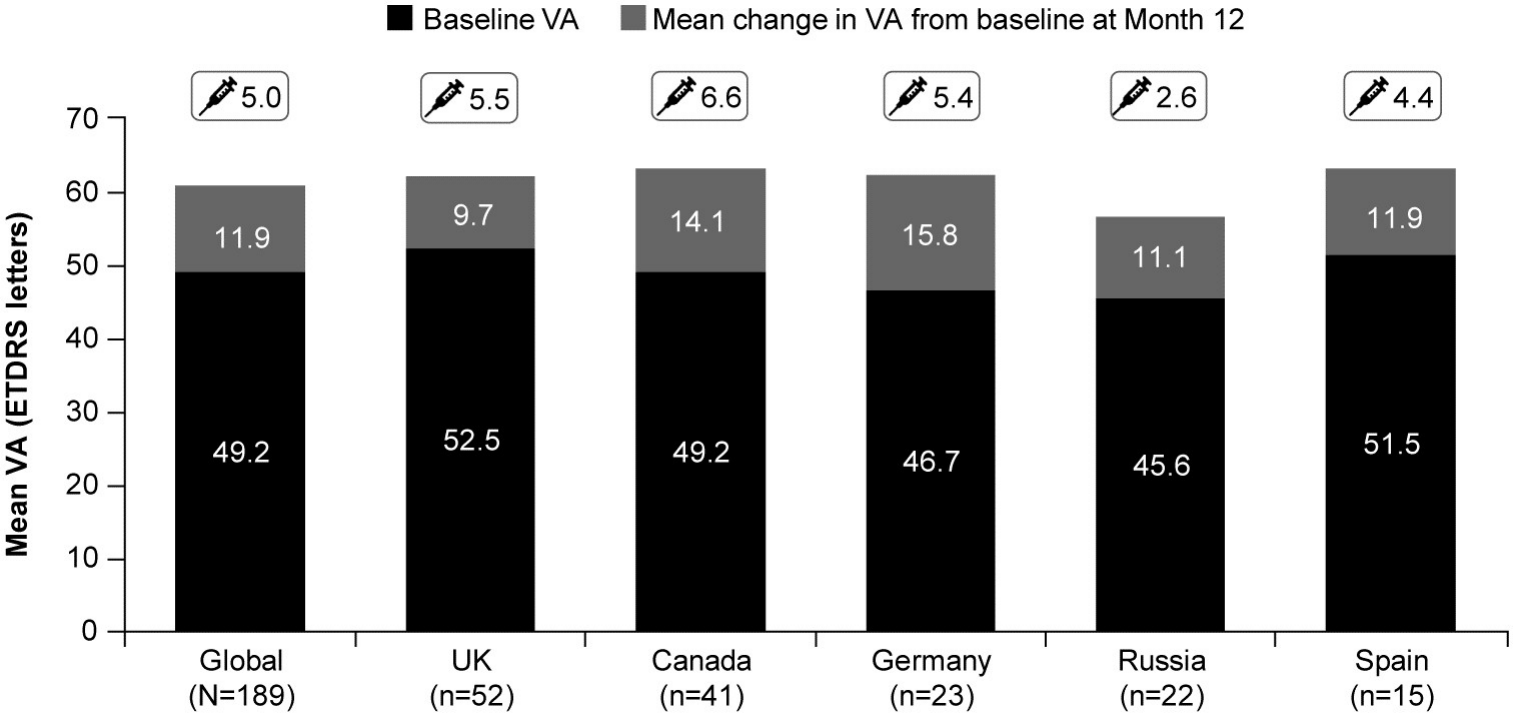
Mean change in VA from baseline at Month 12. Primary treated eye set, defined as the number of evaluable patients with baseline and Month 12 data who have been in the study for at least 365 days. Syringe symbol denotes mean number of injections from baseline to Month 12. There were only 2 patients from India with baseline and Month 12 VA data who have been in the study for at least 365 days. Therefore the mean VA has not been shown for the patients from India. ETDRS, Early treatment diabetic retinopathy study; VA, visual acuity.

Overall, for 189 treatment-naïve BRVO patients the mean (SD) VA at baseline was 49.2 (20.32) letters and the mean (SD) VA gain at Year 1 was 11.9 (17.7) letters in the primary treated eye set (Fig 1). The mean VA gain at Year 1 and the mean number of ranibizumab injections varied across the highest enrolling countries (Fig 1).

### Treatment exposure, visits, and VA gains

**Fig 2:**
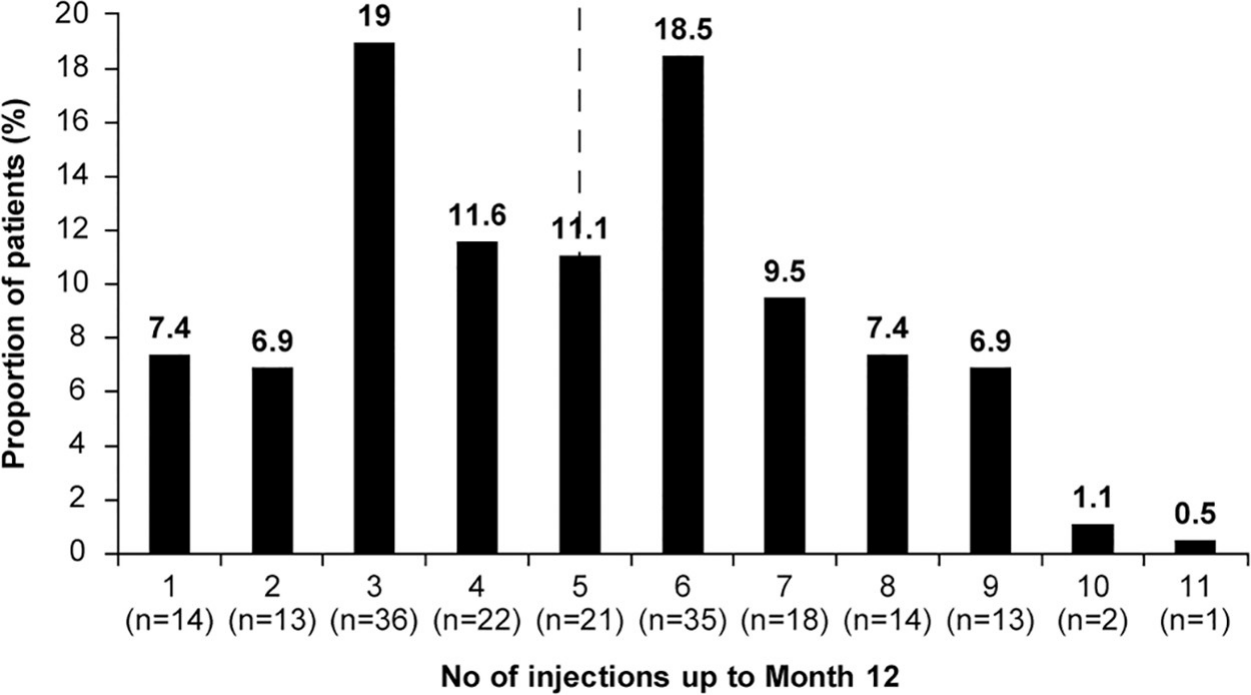
Number of injections over 1 year. Primary treated eye set, defined as number of evaluable patients with baseline and Month 12 data who have been in the study for at least 365 days. Dashed line denotes mean number of injections from baseline to Month 12 in the Global population (n = 189). VA, visual acuity.

The mean (SD) number of ranibizumab injections administered up to Year 1 was 5.0 (2.34), and the mean number of visits was 8.4 (2.91). Overall, 43.9% of patients received 6 or more injections in the first year (Fig 2). Further, it is interesting to note that only 2 patients (1.1%) had received 10 injections and only 1 patient (0.5%) received 11 injections (Fig 2). We consider these patients as extreme outliers.

**Fig 3:**
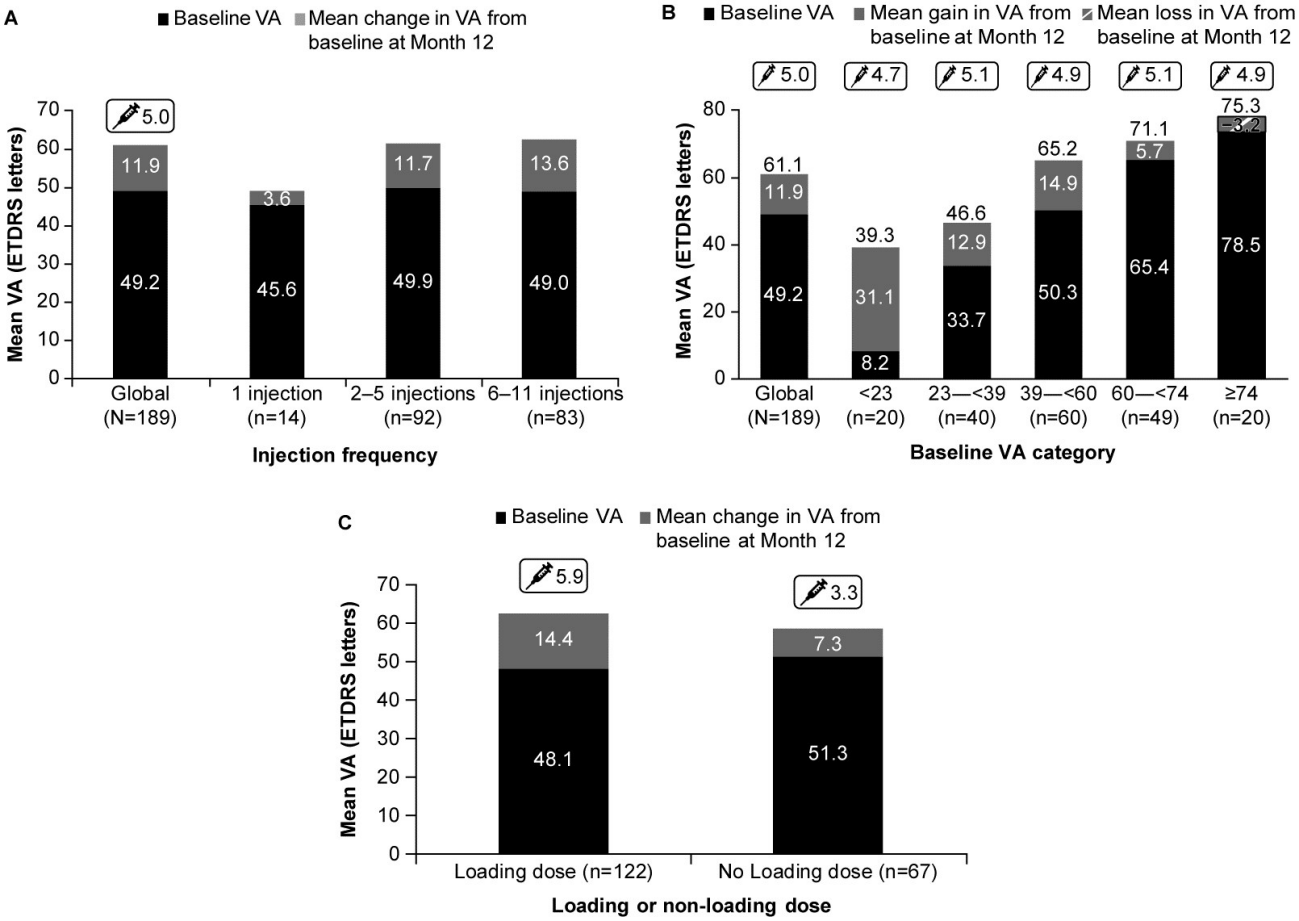
Mean change in VA from baseline at Year 1 by A. injection frequency, B. baseline VA category, and C. in patients who received loading and those who did not. Primary treated eye set, defined as the number of evaluable patients with baseline and Month 12 data who have been in the study for at least 365 days. Syringe symbol denotes mean number of injections from Baseline to Month 12. Loading dose is defined as having 3 ranibizumab injections within the first 100 days of the study. ETDRS, Early treatment diabetic retinopathy study; VA, visual acuity.

The VA gains at Year 1 were greater with increasing treatment frequency. VA gains in patients who received 6–11 injections (n = 83) were 13.6 [20.16] letters, (baseline: 49.0 [21.31]), in those who received 2–5 injections (n = 92) were 11.7 [15.43] letters, (baseline: 49.9 [19.72]) or 1 injection (n = 14) were 3.6 [13.72] letters, (baseline: 45.6 [19.19]) (Fig 3A).

Maximum VA gains (31.1 letters) from baseline at Year 1 were observed in patients with poor baseline VA (<23 ETDRS letters), but the actual VA at Year 1 was higher in patients with higher baseline VA (Fig 3B). All patients (n = 20) with baseline VA ≥74 letters maintained their vision (mean VA [SD] at Year 1 = 75.3 [7.57]) after 1 year of treatment with a mean number of 4.9 injections. The mean number of injections was broadly comparable across the baseline VA categories and ranged from 4.7 to 5.1 (Fig 3B). Patients who received the loading dose of 3 initial consecutive monthly ranibizumab injections (n = 122; 14.4 letters) had numerically higher VA gains than those who did not receive the loading dose (n = 67; 7.3 letters) (Fig 3C).

The mean number of injections in patients who received the loading dose of 3 initial consecutive monthly ranibizumab injections was numerically higher than in patients who did not. Furthermore, patients with baseline VA of ≥73 letters maintained their vision at or close to their starting levels, while patients with baseline VA<73 letters showed VA gains; the number of injections was comparable between the 2 groups (S2 Fig).

At Year 1, of the 189 patients who completed the study, 42.9% (n = 81) of patients treated with ranibizumab had a VA gain of ≥15 letters and VA was maintained at baseline levels in 14.3% (n = 27) of patients (S3 Fig). The proportion of patients with a VA loss of ≥15 letters was low (4.2%; n = 8; S3 Fig).

### Safety

Ocular AEs of the study eye were reported in 7.4% (n = 30) of patients, with cataract (n = 8, 2.0%) being the most common, followed by increased intraocular pressure (n = 3, 0.7%, Table 2). Non-ocular AEs were reported in 9.1% (n = 37) of patients (Table 2). Overall, 5 (1.24%) ocular AEs and 1 (0.25%) non-ocular AE (headache) were suspected by the investigator to be related to the study treatment or ocular injections (S4 Table). There were no ocular AEs or SAEs, 8 (1.98%) non-ocular AEs, and 8 (1.98%) non-ocular SAEs that led to the discontinuation of study treatment.

**Table 2:** Incidence of AEs over 5-years (safety set).

Preferred term, n (%)	Treatment-naïve patients with BRVO N = 405
**Ocular AEs, total**	30 (7.4)
Cataract	8 (2.0)
IOP increased	3 (0.7)
Conjunctivitis	2 (0.5)
Eye pain	2 (0.5)
VA reduced	2 (0.5)
Vitreous floaters	2 (0.5)
Vitreous hemorrhage	2 (0.5)
Conjunctival hemorrhage	2 (0.5)
**Non-ocular AEs, total**	37 (9.1)
Fall	2 (0.5)
Lower respiratory tract infection	2 (0.5)
Ear infection	2 (0.5)
Dehydration	2 (0.5)
Dizziness	2 (0.5)
Cough	2 (0.5)
Influenza	2 (0.5)
Sepsis	2 (0.5)

Ocular and non-ocular AEs ≥2 in number are shown

Indication and pre-treatment status refers to the primary treated eye. Only AEs occurring during the safety observation period are included. Preferred terms are presented by descending order of frequency. A patient with multiple occurrences of an AE was counted only once. A patient with multiple AEs is counted only once in the total row. Patients with a baseline visit date present are included. Data collected until the last recorded follow-up date was used to perform the analyses

AE, adverse event; BRVO, branch retinal vein occlusion; IOP, intraocular pressure; N, total number of patients; n, number of patients; N, total number of patients; VA, visual acuity

Only 1 serious ocular AE (vitreous hemorrhage) was reported in 1 (0.3%) patient; it was considered to be not related to ranibizumab treatment by the investigator. No cases of endophthalmitis or retinal break/detachment were reported. The incidence of non-ocular SAEs was 4.4% (n = 18); dehydration (0.5%) and sepsis (0.5%) were the most frequently reported SAEs, and occurred in 2 patients each (S5 Table).

There were 8 deaths (1.98%) reported over the 5-year duration of the study. There were 6 deaths of unknown cause, and septicemia and blood sepsis were the reasons for deaths in 2 patients. At Year 1 in patients with evaluable baseline and Month 12 VA data available and who had been in the study for at least 365 days (n = 189), the rate of ocular/non-ocular AEs was 3.7% (n = 7)/ 3.2% (n = 6) and serious AEs was 0%/1.1% (n = 2), respectively.

## Discussion

The LUMINOUS study results demonstrate that ranibizumab treatment for 1 year is effective in improving VA outcomes in treatment-naïve patients with BRVO, globally and across different countries. The VA outcomes were better in patients who received adequate treatment at the start of therapy (loading dose) and those who received a higher number of ranibizumab injections. The relative VA gain was higher in patients who had lower baseline VA. However, patients with initially good vision (≥73 letters) maintained their vision and was the group with highest vision at Year 1.

The baseline VA of patients included in this study was a little lower (49.2 letters, primary treated eye set) than the ranibizumab-treated arms of patients included in the randomized, controlled BRAVO (53 letters, randomized set) and BRIGHTER studies (59.5 letters, randomized set) [4, 10]. Although the time from diagnosis to first treatment was a mean of 18 days in this study, the precise information on the actual duration of BRVO prior to presentation is not known in an observational study and suspected to be significantly longer than the BRAVO and BRIGHTER studies. At Year 1, the mean VA gain of 11.9 letters in the LUMINOUS study was lower than that reported in the BRAVO study (18.3 letters). This was expected considering the lower mean number of injections (5.0) in the LUMINOUS study than those reported at 12 months in the BRAVO study (5.7 injections during the 6 month treatment period [Day 0 to Month 5] and 2.7 injections during the 6 month observation period [Months 6–11]) [4]. In the BRIGHTER study, that was conducted across 17 countries worldwide including 455 patients with BRVO, the mean VA at baseline was 59.5 letters and the gain at 24 months was 15.5 letters with a mean of 11.4 injections; VA remained stable between Months 12 and 24 [10].

An important predictor of VA gains with treatment is the patients’ baseline BCVA [21, 24]. In the BRAVO and BRIGHTER studies, it was observed that patients with poor baseline VA had numerically higher VA gains at 12 and 24 months, respectively [4, 10]. Consistent with these observations, numerically higher VA gains were observed in the LUMINOUS study in patients with poor baseline VA BRVO patients with baseline VA of 39–<60 letters demonstrated the best visual gains. However, patients with baseline VA of 60–<74 letters maintained their VA at 12 months with adequate ranibizumab treatment. Furthermore, in the BRAVO and BRIGHTER studies, as well as in the LUMINOUS study, the final VA at 12 or 24 months was lower in patients with poor baseline VA, highlighting the need for early treatment initiation [4, 10]. However, it is important to note that the injection frequency was uniform irrespective of the baseline VA in the LUMINOUS study. The BRIGHTER study also found that disease duration was a factor affecting treatment outcomes; however, it was not possible to confirm this in LUMINOUS [10].

Higher injection frequency in the LUMINOUS study was associated with greater VA gains in the global population across all enrolling countries. The good mean letter gains observed at Year 1 suggests that BRVO patients may not need as many ranibizumab injections as other retinal diseases (such as nAMD and DME) to achieve good visual outcomes [25, 26]. However, it does confirm that adequate dosing must be maintained to achieve optimal BCVA outcomes.

The results of the LUMINOUS study are comparable with other observational studies of ranibizumab in patients with BRVO [21, 27]. In a real-world cross-sectional study, BCVA improved from 44±17.9 to 63±13.7 letters at Year 1, with a mean of 3 injections in the first 6 months and mean of 1 injection in the second half of the first year [27]. Treatment-naïve patients with BRVO from LUMINOUS were treated with ranibizumab in real-world clinical practice settings across various countries with different healthcare systems. In the country-specific analyses, the mean age of patients ranged from 58.1 years (India) to 74.4 years (Canada) suggesting possible country-level differences in the age of disease onset. The mean baseline VA varied among countries with the highest baseline VA observed in India (55.8 letters; n = 28) followed by Spain (54.4 letters; n = 21) and the lowest baseline VA was observed in Canada (49.8 letters; n = 68). Whilst there is no information on disease durations, there was a large variation in median time from diagnosis to first treatment among countries, ranging from 1 and 8 days (Canada and Germany, respectively) to 41 days (Russia). This variation is presumably reflective of differences among healthcare systems and access to treatment and probably also reflects varying differences in disease duration prior to diagnosis in these countries, highlighting the need for education and public awareness. Large gains in VA from baseline (mean VA gains ranged from +9.7 letters in the UK to +15.8 letters in Germany) were observed across all countries, including Russia (+11.1 letters), which had a low mean number of injections (2.6). The low number of injections in Russia may reflect limited patient access to treatment. Despite differences in baseline VA, the final 12-month VA for the UK, Germany, Canada, and Spain were comparable with varying mean numbers of injections (4.4 to 6.6 injections). This observation does reflect a relative level of under treatment compared with the RCTs [10]. Further, in this study, VA improvement in these countries varied from 9.7–15.8 letters. The differences in mean VA gains between Russia and Germany (despite similar mean baseline VA scores of 45.6 and 46.7 letters, respectively) may be due to the greater mean number of injections received by German patients and may reflect differences in the healthcare systems and patient access to treatment in these countries.

Across all treatment-naïve patients with BRVO, the frequency of ocular and non-ocular SAEs and AEs at Year 1 and over 5 years was low. There were no ocular AEs or SAEs, which led to discontinuation of ranibizumab in the study. Ocular and non-ocular AEs related to ranibizumab and/or injection were rare. Overall, the ocular AEs observed in LUMINOUS were consistent with those observed in more restrictive RCT populations and consistent with the well-established safety profile of ranibizumab [4, 5, 7, 10].

The strengths of the study are that it is the first large-scale, prospective, multi-indication, observational, global, post-marketing study of an ocular anti-VEGF agent in a large patient population. Being an observational study, patients included had diverse demographic and baseline characteristics with varying degrees of healthcare access that were more representative of real-world settings. This study provides long-term safety data (over 5 years) with ranibizumab under real-world conditions in a large global population of treatment-naïve patients with BRVO.

The limitations of the LUMINOUS study are similar to other real-world studies and are greatly affected by factors, such as treatment costs, reimbursement policies, ease of access to medicines, geographic availability of enrolled patients, wide demographic inclusion of patients, and sub-optimal treatment due to less strict follow-up measures. Similarly, the variations in median time from diagnosis to first treatment across countries ranging from 1 day in Canada to 41 days in Russia can be attributed to the global differences in access to patient care, health care systems, approvals, reimbursements and administrative processes. In addition, the study had no comparator arm, so the safety and effectiveness of ranibizumab cannot be directly compared with any other intervention or to no intervention.

To conclude, the LUMINOUS study demonstrated clinically meaningful VA improvement with ranibizumab in treatment-naïve patients with BRVO where patients receiving more injections and those with poor baseline VA had numerically higher VA gains. Overall, the safety profile was consistent with what has been observed in previous ranibizumab studies in BRVO and no new safety signals were identified. Results from this study confirms the effectiveness of ranibizumab in BRVO patients in real-world practice, underscores the need for educating patients on the importance of individualized treatment regimen, and may help guide physicians in to effectively manage BRVO patients in clinical practice.

## Supporting information

S1 Fig Country-wise baseline characteristics of treatment-naïve patients with BRVO. A. age, B. mean baseline VA, and C. median time from diagnosis to first ranibizumab treatment. Countries that recruited >20 treatment-naïve BRVO patients *VA for patients with evaluable data at baseline is shown. BRVO, branch retinal vein occlusion; ETDRS, Early Treatment Diabetic Retinopathy Study; VA, visual acuity.
(TIF)

S2 Fig Mean change in VA from baseline at year 1 by baseline VA category. Primary treated eye set, defined as the number of evaluable patients with baseline and Month 12 data who have been in the study for at least 365 days. Syringe symbol denotes mean number of injections from baseline to Month 12. ETDRS, Early treatment diabetic retinopathy study; VA, visual acuity.
(TIF)

S3 Fig Proportion of patients with categorical loss or gain in VA from baseline at year 1. Primary treated eye set, defined as the number of evaluable patients with baseline and Month 12 data who have been in the study for at least 365 days. VA maintained in patients included those with 0 letter loss at Year 1 from baseline. ETDRS, Early treatment diabetic retinopathy study; n, number of patients; VA, visual acuity.
(TIF)

S1 Table List of Independent Ethics Committees (IEC) or Institutional Review Boards (IRB) by study center. (DOCX)

S2 Table Country-wise recruitment of treatment-naïve patients with BRVO. (DOCX)

S3 Table Disposition of treatment-naïve patients with BRVO. (DOCX)

S4 Table Ocular (study eye) and nonocular AEs suspected to be related to ranibizumab treatment and/or ocular injection over a 5-year period (safety set). (DOCX)

S5 Table Incidence of SAEs over a 5-year period (safety set). (DOCX)

S6 Table The list of LUMINOUS study principal investigators. (DOCX)

## References

[1] Rogers S, McIntosh RL, Cheung N, Lim L, Wang JJ, Mitchell P, et al The prevalence of retinal vein occlusion: pooled data from population studies from the United States, Europe, Asia, and Australia. Ophthalmology. 2010;117(2):313–9.e1. 10.1016/j.ophtha.2009.07.017 . 20022117 PMC2945292

[2] Song P, Xu Y, Zha M, Zhang Y, Rudan I. Global epidemiology of retinal vein occlusion: a systematic review and meta-analysis of prevalence, incidence, and risk factors. J Glob Health. 2019;9(1):010427 10.7189/jogh.09.010427 . 31131101 PMC6513508

[3] Pulido JS, Flaxel CJ, Adelman RA, Hyman L, Folk JC, Olsen TW. Retinal Vein Occlusions Preferred Practice Pattern((R)) Guidelines. Ophthalmology. 2016;123(1):P182–208. 10.1016/j.ophtha.2015.10.045 . 26581559

[4] Brown DM, Campochiaro PA, Bhisitkul RB, Ho AC, Gray S, Saroj N, et al Sustained benefits from ranibizumab for macular edema following branch retinal vein occlusion: 12-month outcomes of a phase III study. Ophthalmology. 2011;118(8):1594–602. 10.1016/j.ophtha.2011.02.022 . 21684606

[5] European Medicines Agency. Lucentis^®^ Summary of Product Characteristics. Novartis Pharma AG, Basel, Switzerland. 2016. http://www.ema.europa.eu/docs/en_GB/document_library/EPAR_-_Product_Information/human/000715/WC500043546.pdf (Accessed on December 20, 2019). [Internet].

[6] Food and Drug Administration. Lucentis^®^ Prescribing information. Genentech, Inc., San Francisco, California, USA. 2017. https://www.accessdata.fda.gov/drugsatfda_docs/label/2017/125156s111lbl.pdf (Accessed on December 20, 2019). [Internet].

[7] Heier JS, Campochiaro PA, Yau L, Li Z, Saroj N, Rubio RG, et al Ranibizumab for macular edema due to retinal vein occlusions: long-term follow-up in the HORIZON trial. Ophthalmology. 2012;119(4):802–9. 10.1016/j.ophtha.2011.12.005 . 22301066

[8] Campochiaro PA, Sophie R, Pearlman J, Brown DM, Boyer DS, Heier JS, et al Long-term outcomes in patients with retinal vein occlusion treated with ranibizumab: the RETAIN study. Ophthalmology. 2014;121(1):209–19. Epub 2013/10/12. 10.1016/j.ophtha.2013.08.038 . 24112944

[9] Campochiaro PA, Wykoff CC, Singer M, Johnson R, Marcus D, Yau L, et al Monthly versus as-needed ranibizumab injections in patients with retinal vein occlusion: the SHORE study. Ophthalmology. 2014;121(12):2432–42. 10.1016/j.ophtha.2014.06.011 . 25060610

[10] Tadayoni R, Waldstein SM, Boscia F, Gerding H, Gekkieva M, Barnes E, et al Sustained benefits of ranibizumab with or without laser in branch retinal vein occlusion: 24-month results of the BRIGHTER study. Ophthalmology. 2017;124(12):1778–87. 10.1016/j.ophtha.2017.06.027 . 28807635

[11] Hattenbach LO, Feltgen N, Bertelmann T, Schmitz-Valckenberg S, Berk H, Eter N, et al Head-to-head comparison of ranibizumab PRN versus single-dose dexamethasone for branch retinal vein occlusion (COMRADE-B). Acta Ophthalmol. 2018;96(1):e10–e8. 10.1111/aos.13381 . 28251811

[12] Bandello F, Augustin A, Tufail A, Leaback R. A 12-month, multicenter, parallel group comparison of dexamethasone intravitreal implant versus ranibizumab in branch retinal vein occlusion. Eur J Ophthalmol. 2018;28(6):697–705. 10.1177/1120672117750058 . 29631435 PMC6210573

[13] Novartis data on file DSUR. 2018;(Nov).

[14] Mitchell P, Bandello F, Schmidt-Erfurth U, Lang GE, Massin P, Schlingemann RO, et al The RESTORE study: ranibizumab monotherapy or combined with laser versus laser monotherapy for diabetic macular edema. Ophthalmology. 2011;118(4):615–25. Epub 2011/04/05. 10.1016/j.ophtha.2011.01.031 . 21459215

[15] Prunte C, Fajnkuchen F, Mahmood S, Ricci F, Hatz K, Studnicka J, et al Ranibizumab 0.5 mg treat-and-extend regimen for diabetic macular oedema: the RETAIN study. Br J Ophthalmol. 2016;100(6):787–95. 10.1136/bjophthalmol-2015-307249 . 26453639 PMC4893084

[16] Rosenfeld PJ, Brown DM, Heier JS, Boyer DS, Kaiser PK, Chung CY, et al Ranibizumab for neovascular age-related macular degeneration. N Engl J Med. 2006;355(14):1419–31. Epub 2006/10/06. 10.1056/NEJMoa054481 . 17021318

[17] Tadayoni R, Waldstein SM, Boscia F, Gerding H, Pearce I, Priglinger S, et al Individualized Stabilization Criteria-Driven Ranibizumab versus Laser in Branch Retinal Vein Occlusion: Six-Month Results of BRIGHTER. Ophthalmology. 2016;123(6):1332–44. 10.1016/j.ophtha.2016.02.030 . 27039022

[18] Holz FG, Tadayoni R, Beatty S, Berger A, Cereda MG, Hykin P, et al Key drivers of visual acuity gains in neovascular age-related macular degeneration in real life: findings from the AURA study. Br J Ophthalmol. 2016;100(12):1623–8. 10.1136/bjophthalmol-2015-308166 . 27030279 PMC5256408

[19] Writing Committee for the UK Age-Related Macular Degeneration EMR Users Group. The neovascular age-related macular degeneration database: Multicenter study of 92 976 ranibizumab injections: report 1: Visual acuity. Ophthalmology. 2014;121(5):1092–101. 10.1016/j.ophtha.2013.11.031 . 24461586

[20] Ziemssen F, Feltgen N, Holz FG, Guthoff R, Ringwald A, Bertelmann T, et al Demographics of patients receiving intravitreal anti-VEGF treatment in real-world practice: Healthcare research data versus randomized controlled trials. BMC Ophthalmol. 2017;17(1):7 10.1186/s12886-017-0401-y . 28103831 PMC5244516

[21] Vaz-Pereira S, Marques IP, Matias J, Mira F, Ribeiro L, Flores R. Real-world outcomes of anti-VEGF treatment for retinal vein occlusion in Portugal. Eur J Ophthalmol. 2017:0 10.5301/ejo.5000943 . 28315518

[22] Sharma S, Khan MA, Chaturvedi A, Group R-ESI. Real-life clinical effectiveness of razumab(r) (the world's first biosimilar of ranibizumab) in retinal vein occlusion: A subgroup analysis of the pooled retrospective re-enact study. Ophthalmologica. 2019;241(1):24–31. 10.1159/000488602 . 29945143 PMC6390449

[23] Observe the effectiveness and safety of ranibizumab in real life setting (LUMINOUS) [cited 2019 December 20]. https://clinicaltrials.gov/ct2/show/NCT01318941.

[24] Kim SJ, Yoon YH, Kim HK, Yoon HS, Kang SW, Kim JG, et al Baseline predictors of visual acuity and retinal thickness in patients with retinal vein occlusion. J Korean Med Sci. 2015;30(4):475–82. 10.3346/jkms.2015.30.4.475 . 25829817 PMC4366970

[25] Holz FG, Figueroa MS, Bandello F, Yang Y, Ohji M, Dai H, et al Ranibizumab treatment in treatment-naive neovascular age-related macular degeneration: Results from LUMINOUS, a global real-world study. Retina. 2019 10.1097/IAE.0000000000002670 . 31764612 PMC7447127

[26] Mitchell P, Sheidow TG, Farah ME, Mahmood S, Minnella AM, Eter N, et al Effectiveness and safety of ranibizumab 0.5 mg in treatment-naïve patients with diabetic macular edema: Results from the real-world global LUMINOUS study. Manuscript submitted to PLOS ONE. 2019. 10.1371/journal.pone.0233595 PMC7269267 32492069

[27] Rezar S, Eibenberger K, Buhl W, Georgopoulos M, Schmidt-Erfurth U, Sacu S, et al Anti-VEGF treatment in branch retinal vein occlusion: A real-world experience over 4 years. Acta Ophthalmol. 2015;93(8):719–25. 10.1111/aos.12772 . 26109209

