# Supplementary figures and images for: Real-world outcomes with ranibizumab in branch retinal vein occlusion: The prospective, global, LUMINOUS study
Source: PLoS One. 2020 Jun 18;15(6):e0234739. doi: 10.1371/journal.pone.0234739 (PMC7302470; doi:10.1371/journal.pone.0234739)

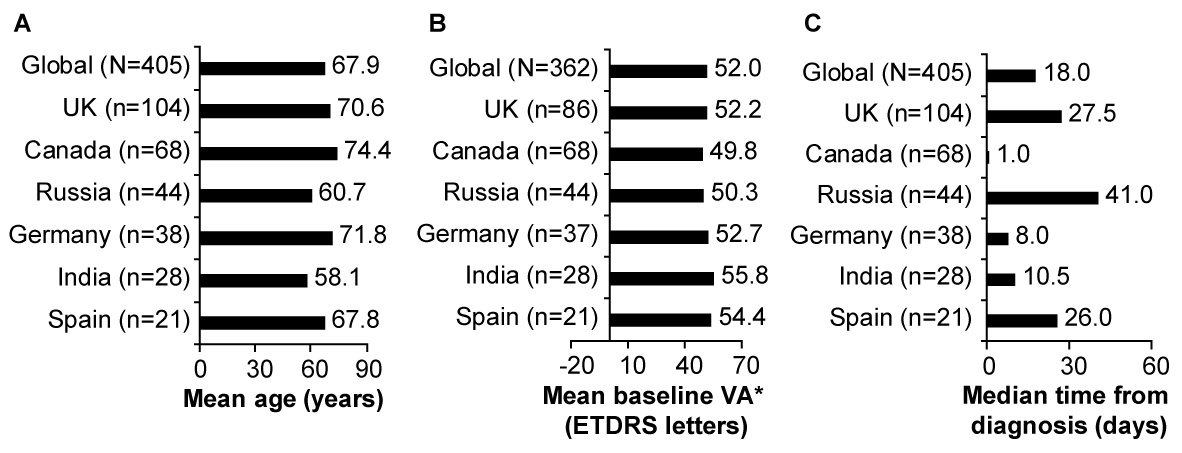

Supplement: S1 Fig — A. age, B. mean baseline VA, and C. median time from diagnosis to first ranibizumab treatment. Countries that recruited >20 treatment-naïve BRVO patients *VA for patients with evaluable data at baseline is shown. BRVO, branch retinal vein occlusion; ETDRS, Early Treatment Diabetic Retinopathy Study; VA, visual acuity. (TIF) [file pone.0234739.s001.tif]

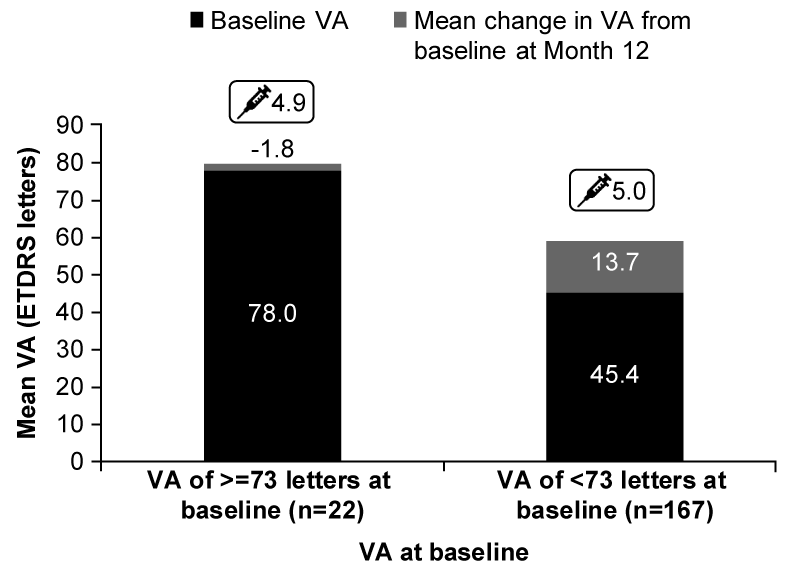

Supplement: S2 Fig — Primary treated eye set, defined as the number of evaluable patients with baseline and Month 12 data who have been in the study for at least 365 days. Syringe symbol denotes mean number of injections from baseline to Month 12. ETDRS, Early treatment diabetic retinopathy study; VA, visual acuity. (TIF) [file pone.0234739.s002.tif]

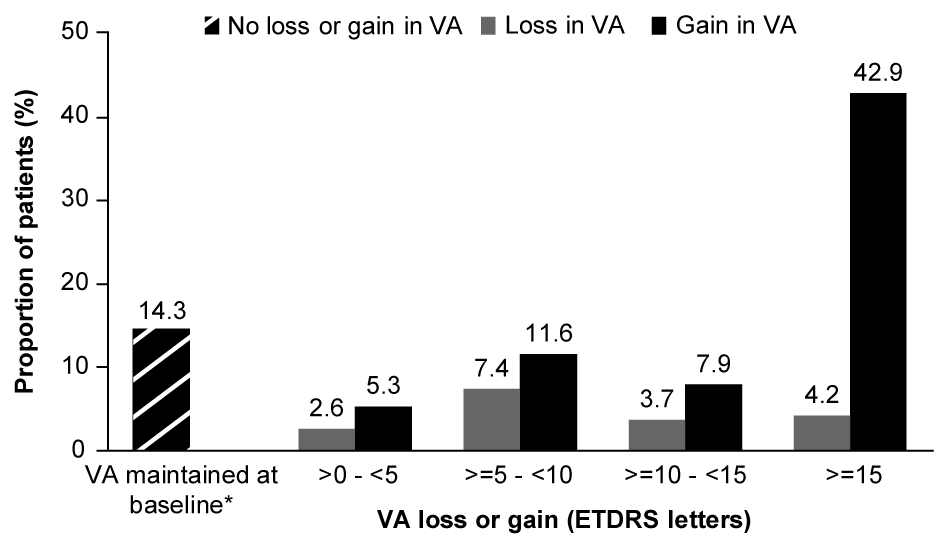

Supplement: S3 Fig — Primary treated eye set, defined as the number of evaluable patients with baseline and Month 12 data who have been in the study for at least 365 days. VA maintained in patients included those with 0 letter loss at Year 1 from baseline. ETDRS, Early treatment diabetic retinopathy study; n, number of patients; VA, visual acuity. (TIF) [file pone.0234739.s003.tif]
