# Supplementary material for: Real-world outcomes with ranibizumab in branch retinal vein occlusion: The prospective, global, LUMINOUS study
Source: PLoS One. 2020 Jun 18;15(6):e0234739. doi: 10.1371/journal.pone.0234739 (PMC7302470; doi:10.1371/journal.pone.0234739)
Supplement: S2 Table — (DOCX) [file pone.0234739.s005.docx]

**S2 Table.** **Country-wise recruitment of treatment-naïve patients with BRVO.**

| **Country** | **Patients*** | **Country** | **Patients*** |
| --- | --- | --- | --- |
| UK | 104 | Malaysia | 4 |
| Canada | 68 | France | 3 |
| Russia | 44 | Greece | 3 |
| Germany | 38 | Ireland | 3 |
| India | 28 | Italy | 3 |
| Spain | 21 | Portugal | 3 |
| Argentina | 20 | Slovakia | 3 |
| Poland | 14 | Brazil | 2 |
| Mexico | 9 | Turkey | 2 |
| Saudi Arabia | 8 | Ukraine | 2 |
| Columbia | 7 | Australia | 1 |
| Egypt | 7 | Ecuador | 1 |
| Czech Republic | 6 | Peru | 1 |
| *Data from enrolled set of patients. Only treatment-naïve patients are shown  BRVO, branch retinal vein occlusion; UK, the United Kingdom | | | |
