# Supplementary material for: Real-world outcomes with ranibizumab in branch retinal vein occlusion: The prospective, global, LUMINOUS study
Source: PLoS One. 2020 Jun 18;15(6):e0234739. doi: 10.1371/journal.pone.0234739 (PMC7302470; doi:10.1371/journal.pone.0234739)
Supplement: S3 Table — (DOCX) [file pone.0234739.s006.docx]

**S3 Table.** **Disposition of treatment-naïve patients with BRVO.**

| **Disposition, n (%)** | **Treatment-naïve patients with BRVO at Year 1**  **N=404*** | **Treatment-naïve patients with BRVO at Year 5**  **N=405^†^** |
| --- | --- | --- |
| Patients with one eye treated in the study (primary treated eye) | 389 (96.23) | 389 ( 96.05) |
| Patients who completed the study | 326 (80.69)** | 247 ( 60.99) |
| Patients who discontinued the study | 78 (19.31) | 158 ( 39.01) |
| Reason for discontinuation |  |  |
| AE | 0 | 0 |
| Abnormal laboratory value | 0 | 0 |
| Abnormal test procedure result | 0 | 0 |
| Unsatisfactory therapeutic effect | 5 (1.24) | 11 ( 2.72) |
| Subject's condition no longer requires study drug | 10 (2.48) | 27 ( 6.67) |
| Subject withdrew consent | 8 (1.98) | 9 ( 2.22) |
| Loss to follow-up | 37 (9.16) | 77 ( 19.01) |
| Administrative problems | 2 (0.49) | 3 ( 0.74) |
| Death | 4 (0.99) | 8 ( 1.98) |
| Pregnancy | 0 | 0 |
| Switched to anti-VEGF other than ranibizumab | 11 (2.72) | 19 ( 4.69) |
| Protocol deviation | 1 (0.25) | 4 ( 0.99) |
| Safety set  *Patients with a baseline visit on or before 1st March 2015 are included. Only data collected up to Month 12 were used to perform the analyses  †Patients with a baseline visit date present are included. Data collected until the last recorded follow-up date was used to perform the analyses  **Number of patients ongoing in the study at Year 1  For treatment-naïve eyes, the date of first on-study injection with ranibizumab was considered the baseline date  AE, adverse event; BRVO; branch retinal vein occlusion; n, number of patients; N, total number of patients;  VEGF, vascular endothelial growth factor | | |
