# Supplementary material for: Real-world outcomes with ranibizumab in branch retinal vein occlusion: The prospective, global, LUMINOUS study
Source: PLoS One. 2020 Jun 18;15(6):e0234739. doi: 10.1371/journal.pone.0234739 (PMC7302470; doi:10.1371/journal.pone.0234739)
Supplement: S4 Table — (DOCX) [file pone.0234739.s007.docx]

**S4 Table.** **Ocular (study eye) and non-ocular AEs suspected to be related to ranibizumab treatment and/or ocular injection over a 5-year period (safety set).**

| **Preferred term, n (%)** | **Treatment-naïve patients with BRVO**  **N=405** |
| --- | --- |
| **Ocular AEs, total** | 5 (1.24) |
| Conjunctival hemorrhage | 2 (0.49) |
| Ocular discomfort | 1 (0.25) |
| Anterior chamber cells | 1 (0.25) |
| Dermatitis allergic | 1 (0.25) |
| Eye pruritus | 1 (0.25) |
| **Non-ocular AEs, total** | 1 (0.25) |
| Procedural headache | 1 (0.25) |
| Indication and pre-treatment status refers to the primary treated eye  Only AEs occurring during the safety observation period are included  Preferred terms are presented within primary system organ class by descending order of frequency of total column  A patient with multiple occurrences of an AE is counted only once in the AE category for that group.  Patients with a baseline visit date present are included.  Data collected until the last recorded follow-up date was used to perform the analyses.  AE, adverse event; BRVO; branch retinal vein occlusion; N, total number of patients; n, number of patients | |
