# Supplementary material for: Real-world outcomes with ranibizumab in branch retinal vein occlusion: The prospective, global, LUMINOUS study
Source: PLoS One. 2020 Jun 18;15(6):e0234739. doi: 10.1371/journal.pone.0234739 (PMC7302470; doi:10.1371/journal.pone.0234739)
Supplement: S5 Table — (DOCX) [file pone.0234739.s008.docx]

**S5 Table.** **Incidence of SAEs over a 5-year period (safety set).**

| **Preferred term, n (%)** | **Treatment-naïve patients with BRVO**  **N=405** |
| --- | --- |
| Ocular SAEs, total | 1 (0.3) |
| Vitreous hemorrhage | 1 (0.3) |
| Non-ocular SAEs, total | 18 (4.4) |
| Dehydration | 2 (0.5) |
| Sepsis | 2 (0.5) |
| Pneumonia | 1 (0.3) |
| Cerebrovascular accident | 1 (0.3) |
| Venous thrombosis limb | 1 (0.3) |
| Staphylococcal sepsis | 1 (0.3) |
| Intracardiac mass | 1 (0.3) |
| Indication and pre-treatment status refers to the primary treated eye. Only SAEs occurring during the safety observation period are included. Preferred terms are presented by descending order of frequency. A patient with multiple occurrences of a SAE was counted once per preferred term. A patient with multiple SAEs is counted only once in the total row. Patients with a baseline visit date present are included. Data collected until the last recorded follow-up date was used to perform the analyses  BRVO, branch retinal vein occlusion; N, total number of patients; n, number of patients; SAE, serious adverse events | |
